# Supplementary material for: Reliability and Validity Estimate of the Pro-Inflammatory/Anti-Inflammatory Food Intake Score in South American Pediatric Population: SAYCARE Study
Source: Int J Environ Res Public Health. 2023 Jan 6;20(2):1038. doi: 10.3390/ijerph20021038 (PMC9859242; doi:10.3390/ijerph20021038)
Supplement: Supplementary file 1 [file ijerph-20-01038-s001.zip › ijerph-2015306-SI.pdf]

Supplementary Material

Supplementary Table S1. Different types of fruits and vegetables reported at the 24-hour dietary recall.

Fruits	Vegetables
1. Avocado	1. Asparagus
2. Apple	2. Baked potato
3. Banana	3. Beetroot
4. Fruit Salad	4. Black beans
5. Green grape	5. Broccoli
6. Mango	6. Brown beans
7. Melon	7. Cabbage
8. Natural Carrot Juice	8. Cabbage (green col)
9. Natural coconut water	9. Carrot
10. Natural lemon juice	10. Chard
11. Natural Orange Juice	11. Chayote
12. Orange	12. Corn on the cob
13. Papaya	13. Cucumber
14. Papaya "*Formosa*"	14. Eggplant
15. Peach	15. Garlic
16. Pear	16. Ginger
17. Plum	17. Lentil
18. Purple grape	18. Lettuce
19. Strawberry	19. Maize, corn
20. Tangerine	20. Mushroom
21. Tomato	21. Onion
22. Watermelon	22. Peas
	23. Pepper
	24. Pumpkin
	25. Roasted potatoes
	26. Spinach
	27. Sweet potato
	28. Vegetable soup
	29. White beans
	30. Zucchini

Supplementary Table S2. Characteristics of children and adolescents from a sub-sample for reliability and validity analyses of the SAYCARE Study.

	Reliability				Criterion Validity				Criterion Validity			
	Children (n = 149)		Adolescent s (n=102)		Children (n = 257)		Adolescents (n=146)		Children (n = 69)		Adolescents (n=33)	
Independent variables	% or Mean	(95% CI)	% or Mean	(95% CI)	% or Mean	(95% CI)	% or Mean	(95% CI)	% or Mean	(95% CI)	% or Mean	(95% CI)
Age	6.8	(6.4; 7.2)	14.5	(14.0; 14.9)	6.7	(6.4; 7.0)	14.5	(14.1; 14.9)	8.2	(7.7; 8.6)	15.1	(14.4; 15.8)
Biological Sex												
Female	54.4	(46.3; 62.2)	55.8	(46.0; 65.3)	56.4	(50.3; 62.4)	50.7	(42.5; 58.8)	47.8	(36.2; 59.7)	48.5	(31.6; 65.7)
Male	45.3	(37.8; 53.7)	44.1	(34.7; 54.0)	43.6	(37.6; 49.7)	49.3	(41.2; 57.4)	52.2	(40.3; 63.8)	51.5	(34.3; 68.4)
Type of School												
Public	58.4	(50.3; 66.1)	31.4	(23.0; 41.1)	51.4	(45.2; 57.5)	43.8	(36.0; 52.0)	46.4	(34.8; 58.3)	27.3	(23.0; 41.1)
Private	41.6	(33.9; 49.7)	68.2	(58.8; 77.0)	48.7	(42.5; 54.8)	56.1	(48.0; 64.1)	52.1	(41.7; 65.2)	72.7	(54.6; 85.5)
Nutritional Status												
Thinness	6.7	(3.6; 12.1)	3.9	(1.5; 10.1)	5.8	(3.5; 9.5)	2.7	(1.0; 7.1)	2.9	(0.4; 19.3)		
Normal Weight	67.1	(59.1; 74.2)	62.7	(52.9; 71.7)	70.4	(64.5; 75.7)	65.1	(56.9; 72.4)	73.6	(55.7; 86.0)	66.7	(48.5; 80.9)
Overweight	17.4	(12.1; 24.5)	26.5	(18.7; 36.0)	13.2	(9.6; 18.0)	24	(17.7; 31.6)	17.6	(7.9; 34.9)	33.3	(19.1; 51.5)
Obesity	8.7	(5.11; 14.5)	6.8	(3.3; 13.8)	10.5	(7.3; 14.9)	8.2	(4.7; 14.0)	5.9	(1.4; 21.6)		
Waist circumference (cm)	57.3	(55.9; 58.8)	74.1	(72.4; 75.7)	57.7	(56.7; 58.7)	74.3	(72.7; 75.9)	59.1	(57.1; 61.1)	74.6	(72.4; 76.8)
hs-CRP (mg/dl)									0.6	(0.38; 0.82)	0.32	(0.2; 0.6)

95%CI= 95% confidence interval; BMI= Body Mass Index; WC= Waist circumference; hs-CRP= High sensitive C-reactive protein.

Supplementary Table S3. Reliability of the PAIFIS in grams per day in a sub-sample of the SAYCARE Study.

	Children						Adolescents					
		FFQ1		FFQ2				FFQ1		FFQ2		
	n	Mean	(95% CI)	Mean	(95% CI)	ρ*	n	Mean	(95% CI)	Mean	(95% CI)	ρ*
Pro - Inflammatory food group												
**Sugar-Sweetened Beverages**	146	274.1	(212.1; 336.1)	253.9	(193.0; 314.9)	**0.66**	97	346.7	(276.9; 416.5)	297.7	(236.7; 358.7)	**0.59**
**Processed Meat**	126	23	(19.7; 26.3)	21.8	(18.8; 24.8)	**0.63**	93	28.7	(23.1; 34.2)	34.3	(26.2; 42.3)	**0.54**
**Red meat**	141	52	(44.2; 59.8)	58.8	(48.1; 69.5)	**0.54**	93	52.2	(35.4; 69.0)	55	(41.9; 68.4)	**0.57**
**Candies**	149	175.9	(146.4; 205.4)	154.2	(130.0; 178.3)	**0.51**	102	165.8	(139.4; 192.1)	177.5	(137.9; 217.0)	**0.31**
**Snacks**	136	35.6	(29.7; 41.5)	29.8	(25.2; 34.4)	**0.56**	96	35.1	(28.9; 41.4)	41	(30.1; 51.8)	**0.42**
Anti - Inflammatory food group												
**Fruits**	149	279.8	(242.8; 316.8)	239.1	(205.5; 272.6)	**0.58**	97	353.5	(272.0; 434.9)	298.3	(240.6; 356.0)	**0.53**
**Vegetables**	133	50.1	(41.5; 58.6)	49.2	(39.1; 59.4)	**0.59**	75	62.5	(40.1; 84.9)	50.8	(37.1; 64.5)	**0.47**
Sum groups												
**Pro-Inflammatory**	149	557.2	(489.1; 625.3)	504.2	(435.2; 573.3)	**0.57**	102	618	(539.9 696.1)	580.5	(496.6; 664.5)	**0.52**
**Anti - Inflammatory**	149	319.1	(278.7; 359.5)	278.5	(241.7; 315.3)	**0.63**	97	404.4	(315.7; 493.2)	337	(273.9; 401.3)	**0.55**
**PAIFIS = Σ AI - Σ PI**	149	238.1	(166.3; 309.9)	225.7	(158.3; 293.2)	**0.69**	102	223.3	(121.0; 325.6)	259.5	(176.4; 342.7)	**0.61**

95%CI= 95% confidence interval; ρ= Spearman rho coefficient; PCV= proportional change attributable to region-level variance; R= regression coefficient; (ΣPI) = Sum Pro-inflammatory food group= ΣAI, Sum Anti-Inflammatory food group; PAIFIS= Σ AI - Σ PI.

Supplementary Table S4. Convergent validity of the PAIFIS in grams per day in a sub-sample of the SAYCARE Study.

Children	FFQ1			R24hrs				
	n	Mean	(95% CI)	Mean	(95% CI)	ρ	*R**	PCV*
Pro - Inflammatory food group								
Sugar-Sweetened Beverages	224	276.51	(229.44; 323.59)	575.34	(518.9; 631.78)	0.22	-	-
Processed Meat	257	20.51	(16.64; 24.38)	23.34	(19.35; 27.33)	**0.37**	-	-
Red meat	257	49.3	(42.52; 56.08)	56.9	(48.90; 63.13)	**0.31**	-	-
Candies	257	161.43	(140.42; 182.44)	107.05	(93.68; 120.42)	**0.4**	-	-
Snacks	257	26.21	(22.74; 29.67)	43.95	(36.15; 51.7)	0.28	-	-
Anti - Inflammatory food group								
Fruits	232	41.56	(34.50; 48.63)	129.47	(115.25; 143.69)	0.22	-	-
Vegetables	228	310.46	(278.61; 342.31)	210.01	(188.60; 231.42)	0.23	-	-
Sum groups							-	-
Pro-Inflammatory	257	514.27	(464.90; 563.63)	731.82	(668.29; 795.350)	**0.36**	-	-
Anti-Inflammatory	257	341.14	(309.91; 372.39)	303.19	(273.91; 332.48)	0.20	-	-
PAIFIS	257	173.12	(121.09; 225.16)	428.45	(360.79; 496.47)	0.23	0.32	43.34

Adolescents	FFQ1			R24hrs				
	n	Mean	(95% CI)	Mean	(95% CI)	ρ	*R**	PCV*
Pro - Inflammatory food group								
Sugar-Sweetened Beverages	133	371.6	(312.09; 431.10)	655.45	(572.63; 738.27)	0.15	-	-
Processed Meat	95	42.51	(32.00; 53.01)	51.18	(41.51; 60.86)	0.09	-	-
Red meat	108	65.11	(48.70; 81.52)	74.22	(64.40; 84.05)	0.24	-	-
Candies	136	181.69	(151.92; 211.47)	80.97	(68.33; 93.60)	0.18	-	-
Snacks	81	34.19	(26.91; 41.47)	105.9	(84.67; 127.13)	0.25	-	-
Anti - Inflammatory food group								
Fruits	110	242.33	(165.43; 319.23)	129.85	(105.54; 154.17)	0.16	-	-
Vegetables	121	72.56	(55.01; 90.11)	68.44	(56.52; 80.35)	0.17	-	-
Sum groups							-	-
Pro-Inflammatory	146	654	(588.39; 719.60)	819.47	(727.82; 911.12)	0.26	-	-
Anti-Inflammatory	146	362.44	(287.18; 437.71)	223.35	(187.50; 259.20)	0.29	-	-
PAIFIS	146	291.56	(191.27; 391.84)	596.12	(505.97; 686.28)	0.16	0.24	15.45

95%CI= 95% confidence interval; ρ= Spearman rho coefficient; PCV= proportional change attributable to region-level variance; R= regression coefficient; (ΣPI) = Sum Pro-inflammatory food group= ΣAI, Sum Anti-Inflammatory food group; PAIFIS= Σ AI - Σ PI.

* coefficient from multilevel regression models after adjusted for center; type of school, sex, age, and total energy intake.

Supplementary Table S5. Validity criterion of the PAIFIS in grams per day with hs-CRP in a sub-sample of the SAYCARE Study.

		ΣPI (g/day)	(ΣAI) (g/day)	PAIFIS (g/day)
Children (n=69)	Mean ± SD	447.70±30.63	314.70±39.44	132.97±51.29
	(95%CI)	(386.56 - 508.80)	(236.01 - 393.40)	(30.62 - 235.33)
	**ρ**	0.01	0.03	-0.03
	R*	-	-	0.48
	**PCV***	-	-	43.94
Adolescents (n=33)	Mean ± SD	536.75 ± 56.57	291.33 ± 55.78	245.43 ± 67.58
	(95%CI)	(421.53 - 651.98)	(177.73 - 404.94)	(107.77 - 383.08)
	**ρ**	0.16	0.07	0.24
	R*	-	-	0.33
	**PCV***	-	-	61.70

hs-CRP= High sensitive C-reactive protein; 95%CI= 95% confidence interval; **ρ**= Spearman rho coefficient; PCV= proportional change attributable to region-level variance; R= regression coefficient; (ΣPI) = Sum Pro-inflammatory food group= ΣAI, Sum Anti-Inflammatory food group; PAIFIS= Σ AI - Σ PI.

*coefficient from multilevel regression models after adjusted for center; type of school, sex, age, and total energy intake.
